# IRX-related homeobox gene *MKX* is a novel oncogene in acute myeloid leukemia

**DOI:** 10.1371/journal.pone.0315196

**Published:** 2024-12-17

**Authors:** Stefan Nagel, Corinna Meyer, Claudia Pommerenke

**Affiliations:** Dept. of Human and Animal Cell Lines, Leibniz-Institute DSMZ, Braunschweig, Germany; Duke University Medical Center: Duke University Hospital, UNITED STATES OF AMERICA

## Abstract

Homeobox genes encode transcription factors which organize differentiation processes in all tissue types including the hematopoietic compartment. Recently, we have reported physiological expression of TALE-class homeobox gene *IRX1* in early myelopoiesis restricted to the megakaryocyte-erythroid-progenitor stage and in early B-cell development to the pro-B-cell stage. In contrast, sister homeobox genes *IRX2*, *IRX3* and *IRX5* are aberrantly activated in the corresponding malignancies acute myeloid leukemia (AML) and B-cell progenitor acute lymphoid leukemia. Here, we examined the role of IRX-related homeobox gene *MKX* (also termed *IRXL1* or *mohawk*) in normal and malignant hematopoiesis. Screening of public datasets revealed silent *MKX* in normal myelopoiesis and B-cell differentiation, and aberrant expression in subsets of AML and multiple myeloma (MM) cell lines and patients. To investigate its dysregulation and oncogenic function we used AML cell line OCI-AML3 as model which strongly expressed *MKX* at both RNA and protein levels. We found that IRX5, JUNB and NFkB activated *MKX* in this cell line, while downregulated GATA2 and STAT5 inhibited its expression. MKX downstream analysis was conducted by siRNA-mediated knockdown and RNA-sequencing in OCI-AML3, and by comparative expression profiling analysis of a public dataset from MM patients. Analysis of these data revealed activation of *CCL2* which in turn promoted proliferation. Furthermore, MKX upregulated *SESN3* and downregulated *BCL2L11*, which may together underlie decreased etoposide-induced apoptosis. Finally, myeloid differentiation genes *CEBPD* and *GATA2* were respectively up- and downregulated by MKX. Taken together, our study identified *MKX* as novel aberrantly expressed homeobox gene in AML and MM, highlighting the function of *IRX1* in normal myelopoiesis and B-cell development, and of IRX-related genes in corresponding malignancies. Our data merit further investigation of *MKX* and its deregulated target genes to serve as novel markers and/or potential therapeutic targets in AML patient subsets.

## Introduction

The hematopoietic system is divided into the myeloid and lymphoid lineages, generating all blood and immune cells lifelong, forming a highly proliferative tissue. On the other hand, reflecting this high proliferative capacity, hematopoietic cells are prone to malignant transformation. Accordingly, specific genetic alterations, like chromosomal and genomic rearrangements or gene mutations in hematopoietic stem and progenitor cells, often underlie aberrant gene activities and the development of leukemia and lymphoma. Subtype-specific aberrations serve as diagnostic markers and may comprise therapeutic targets [1–4]. Therefore, identification of novel mutated or deregulated genes extends the potential for the clinical treatment.

Regulation of developmental processes in hematopoiesis is primarily orchestrated at the transcriptional level [5–10]. Accordingly, many hematopoietic malignancies display mutations and/or deregulations of transcription factors (TFs). Therefore, analysis of TF-encoding genes may reveal novel pathological mechanisms and clinical tracks. The human genome contains about 1.600 TF-encoding genes [11]. These genes and factors are classified according to sequence and structure of their DNA-binding domains. Homeobox genes encode developmental TFs, sharing a conserved homeodomain which contains three alpha-helices connected by two loops [12–14]. TALE-class homeodomain factors share a three amino acid insertion within the first loop of their homeodomain, representing a unique feature [13]. Due to their basic impact in cell and tissue differentiation, homeobox genes are frequently deregulated in cancer including leukemia/lymphoma [15–17]. PBX1 is a well-known member of the TALE-class and physiologically expressed in early stem and progenitor cells but silent in developing B-cells [18]. However, in B-cell progenitor acute lymphoid leukemia (BCP-ALL) *PBX1* is part of the fusion gene *TCF3*::*PBX1*, and in Hodgkin lymphoma *PBX1* is aberrantly activated, demonstrating that deregulated *PBX1* acts as an oncogene in B-cell malignancies [18, 19].

In the past, we have reported about selected groups of TFs and described their gene activities in the hematopoietic compartment. The obtained TF patterns were called codes. Accordingly, we have established the so-called “TALE-code” which describes physiological activities of TALE-class homeobox genes in all progenitors and mature cell types of myeloid and lymphoid hematopoiesis [20, 21]. This code informs our understanding of developmental gene regulation in the hematopoietic compartment, and helps identification of deregulated TALE-class homeobox genes in corresponding leukemias and lymphomas [10, 18]. This schema helped disclose that TALE-class homeobox gene *IRX1* is physiologically expressed in specific lymphoid and myeloid progenitors, namely pro-B-cells and megakaryocyte-erythroid-progenitors (MEPs), respectively [20, 21]. On the other hand, the closely related homeobox genes *IRX2*, *IRX3* and *IRX5* are aberrantly expressed in subsets of BCP-ALL and acute myeloid leukemia (AML) patients and cell lines [20–23]. These findings indicate that in the normal course of lymphoid and myeloid development *IRX1* performs specific gene regulations in these progenitors and that continued differentiation and maturation are contingent upon its silencing, while aberrant activities of related IRX-genes disturb these processes [10, 20–22].

Here, we analyzed TALE-class homeobox gene *MKX* (also termed *IRXL1* or *mohawk*) which plays a physiological role in tendon development and is closely related to the hematopoietically expressed *IRX1* gene [13, 24]. *MKX* was found to be silent in normal hematopoiesis and aberrantly expressed in specific hematopoietic malignancies. The identified *MKX*-expressing AML cell line OCI-AML3 was used as a model to investigate the role of this TALE-class homeobox gene in leukemogenesis.

## Materials and methods

### Bioinformatic analyses of gene expression profiling and RNA-seq data

Gene expression data for normal cell types and patients were obtained from Gene Expression Omnibus (GEO, www.ncbi.nlm.nih.gov). We analyzed myelopoietic cells using datasets GSE42519 and GSE19599, AML patients using datasets GSE15434, GSE21261, GSE14468 and GSE35784, and MM patients using GSE19554 and GSE57317 [25–31]. The associated online tool GEO2R provided comparison of patient subsets, revealing the top 250 significant differentially expressed genes [32]. Furthermore, we analyzed RNA-seq data of normal cell types from The Human Protein Atlas (www.proteinatlas.org), and of 100 leukemia/lymphoma cell lines termed LL-100 using dataset E-MTAB-7721 obtained via ArrayExpress (www.ebi.ac.uk/arrayexpress) [33, 34]. Analysis of LL-100 RNA-seq data was performed via the online tool DSMZCellDive [35]. RNA-sequencing data from siRNA-treated OCI-AML3 cells (performed in triplicate) were generated at Eurofins MWG (Ebersberg, Germany). Sample libraries for control and treated cells were prepared with the strand-specific cDNA library and sequenced 2x150bp on the Illumina NovaSeq 6000 platform by Eurofins Genomics (INVIEW Transcriptome, Ebersberg, Germany), aiming a minimum of 30M reads per sample with an insert size of >150bp. Trimming and quality control of the sequencing reads were performed via fastp and quantification of reads via salmon on the reference human gene code GRCh38, version 37 [36, 37]. Finally, data were analyzed using DESeq2 and R/Bioconductor to retrieve normalized count data [38]. Sequencing data are available from BioStudies (www.ebi.ac.uk/biostudies) via BSST1315.

### Cell lines and treatments

Cell lines are held at the DSMZ (Braunschweig, Germany) and were cultivated as described (www.DSMZ.de). All cell lines had been authenticated and tested negative for mycoplasma infection. Inhibition of gene expression levels within cell lines was performed using gene specific siRNA oligonucleotides with reference to AllStars negative Control siRNA (siCTR), all obtained from Qiagen (Hilden, Germany). Overexpression studies were performed using commercial cDNA-constructs cloned into expression vector pCMV6 (Thermo Fisher Scientific, Darmstadt, Germany). SiRNAs (100 pmol) and plasmid-DNA (2 μg) were transfected into 1x10^6^ cells by electroporation using the EPI-2500 impulse generator (Fischer, Heidelberg, Germany) at 350 V for 10 ms. Electroporated cells were harvested after 20 h cultivation. RNA-sequencing analysis was performed in triplicate of OCI-AML3 cells treated by siRNA-mediated knockdown at Eurofins MWG (Konstanz, Germany). We used RNAeasy for RNA extraction (Qiagen), and Bioanalyzer for quality control (Agilent Technologies, Santa Clara, USA). Additional cell treatments were performed using 20 ng/ml recombinant BMP2 or TNFSF11 (R & D Systems, Wiesbaden, Germany), 100 μM azacytidine (AZA), 10 μM dorsomorphin, 14 μM NFkB-inhibitor, and 100 μM etoposide (Sigma-Aldrich, Taufkirchen, Germany) for 20 h. For functional testing, treated cells were analyzed using the IncuCyte S3 Live-Cell Imaging Analysis System and the Cell-by-Cell analysis software (Sartorius, Göttingen, Germany). For detection of apoptotic cells, we additionally used the IncuCyte Caspase-3/7 Green Apoptosis Assay diluted at 1:2000 (Sartorius). Live-cell imaging experiments were performed twice with fourfold parallel tests.

### Polymerase chain-reaction (PCR) analyses

Total RNA was extracted from cultivated cell lines using TRIzol reagent (Thermo Fisher Scientific). Primary human total RNA derived from B-cells, T-cells, monocytes, peripheral blood mononuclear cells (PBMC), brain and prostate were purchased from Biochain/BioCat (Heidelberg, Germany). cDNA was synthesized using 1 μg RNA, random priming and Superscript II (Thermo Fisher Scientific). Real time quantitative (RQ)-PCR analysis was performed using the 7500 Real-time System and commercial buffer and primer sets (Thermo Fisher Scientific). For normalization of expression levels, we quantified the transcripts of TATA box binding protein (*TBP*). Quantitative analyses were performed as biological replicates and measured in triplicate. Standard deviations are presented in the figures as error bars. Statistical significance was assessed by Student´s T-Test (two-tailed) and the resultant p-values indicated by asterisks (* p<0.05, ** p<0.01, *** p<0.001, n.s. not significant).

### Protein analysis

Western blots were generated by the semi-dry method. Protein lysates from cell lines were prepared using SIGMAFast protease inhibitor cocktail (Sigma-Aldrich). Proteins were transferred onto nitrocellulose membranes (Bio-Rad, München, Germany) and blocked with 5% dry milk powder dissolved in phosphate-buffered-saline buffer (PBS). The following antibodies were used: alpha-Tubulin (Sigma-Aldrich, #T6199), and MKX (Santa Cruz Biotechnology, Heidelberg, Germany # sc-515878). For loading control, blots were reversibly stained with Poinceau (Sigma-Aldrich) and detection of alpha-Tubulin (TUBA) performed thereafter. Secondary antibodies were linked to peroxidase for detection by Western-Lightning-ECL (Perkin Elmer, Waltham, MA, USA). Documentation was performed using the digital system ChemoStar Imager (INTAS, Göttingen, Germany). CCL2 protein was quantified in cell line supernatants by Enzyme-Linked Immunosorbant Assay (ELISA) using Human CCL2 Quantikine ELISA Kit (R & D Systems, #DCP00). Supernatants were harvested from 1x10^6^ cells growing in 1 ml medium for 24h and stored at -20°C.

## Results

### *MKX* expression in normal tissues and hematopoietic malignancies

TALE-class homeobox gene *IRX1* is physiologically expressed at the MEP stage of early myelopoiesis and at the pro-B-cell stage of early B-cell differentiation, while the closely related homeobox gene *MKX* was found to be absent in these cells following analysis of public gene expression profiling datasets GSE42519 and GSE19599 (S1 Fig) [20, 21]. Furthermore, RNA-seq data from the Human Protein Atlas discounted *MKX* expression in mature myeloid and lymphoid hematopoietic cells, but prominent activity in brain, prostate and ovary (S1 Fig). Thus, in contrast to *IRX1*, *MKX* is silent in normal hematopoietic cells including MEPs and pro-B-cells.

**Fig 1:**
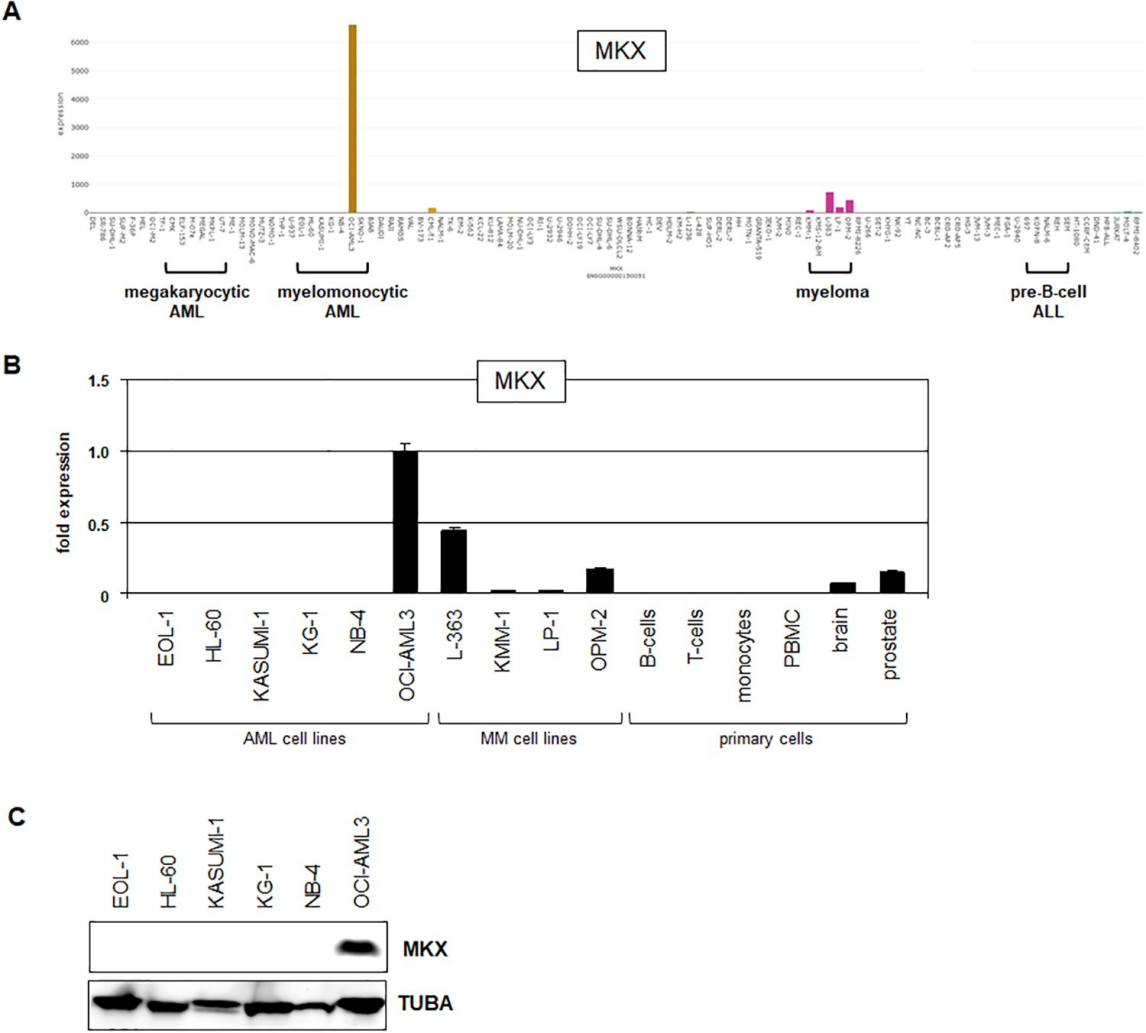
MKX expression analysis. (A) *MKX* expression in 100 leukemia/lymphoma cell lines using RNA-seq data. The following entities are indicated: megakaryocytic AML, myelomonocytic AML, myeloma, and pre-B-cell ALL. MKX-positive cell lines are OCI-AML3 (AML), and KMM-1, L-363, LP-1 and OPM-2 (myeloma). (B) RQ-PCR analysis of *MKX* in AML and MM cell lines, and selected primary cells. The expression level of OCI-AML3 was set as 1 while the remaining samples are related to that value. (C) Western blot analysis of MKX in AML cell lines. TUBA served as loading control.

Recently, we sequenced RNA from 100 leukemia/lymphoma cell lines, covering diverse hematopoietic malignancies, derived from myeloid and lymphoid lineages [34]. Screening of these RNA-seq data demonstrated prominent *MKX* expression in AML cell line OCI-AML3, low levels in multiple myeloma (MM) cell lines KMM-1, L-363, LP-1 and OPM-2, while cell lines derived from other entities including BCP-ALL tested negative (Fig 1A). Analysis of patients with corresponding malignancies using public gene expression profiling datasets showed *MKX* activity in about 1% of AML, and 10% of MM patients (S2 Fig). Thus, we identified aberrantly expressed *MKX* in subsets of AML and MM cell lines and patients, albeit at different frequencies.

RQ-PCR analysis confirmed enhanced MKX RNA expression in OCI-AML3, lower transcript levels in MM cell lines, and absent gene activity in AML control cell lines and primary immune and blood cells, including B-cells, T-cells, monocytes and peripheral blood mononuclear cells (Fig 1B). OCI-AML3 expressed even higher *MKX* transcript levels as compared to primary brain and prostate samples, supporting aberrant activation in this AML cell line (Fig 1B). Finally, Western blot analysis showed MKX expression in OCI-AML3 at the protein level, endorsing this cell line as a suitable model for *MKX* in AML (Fig 1C). Of note, screening of 32 AML cell lines using public gene expression profiling dataset GSE59808 confirmed *MKX* activity in OCI-AML3 while the remaining cell lines tested negative (S3 Fig).

Taken together, *MKX* is aberrantly expressed in subsets of AML and MM patients and cell lines thereby highlighting its likely oncogenic role in these malignancies. Due to prominent *MKX* expression in OCI-AML3, we used this cell line as a model to investigate deregulation and function of this TALE-class homeobox gene in AML.

### Regulation of *MKX* expression in OCI-AML3

**Fig 2:**
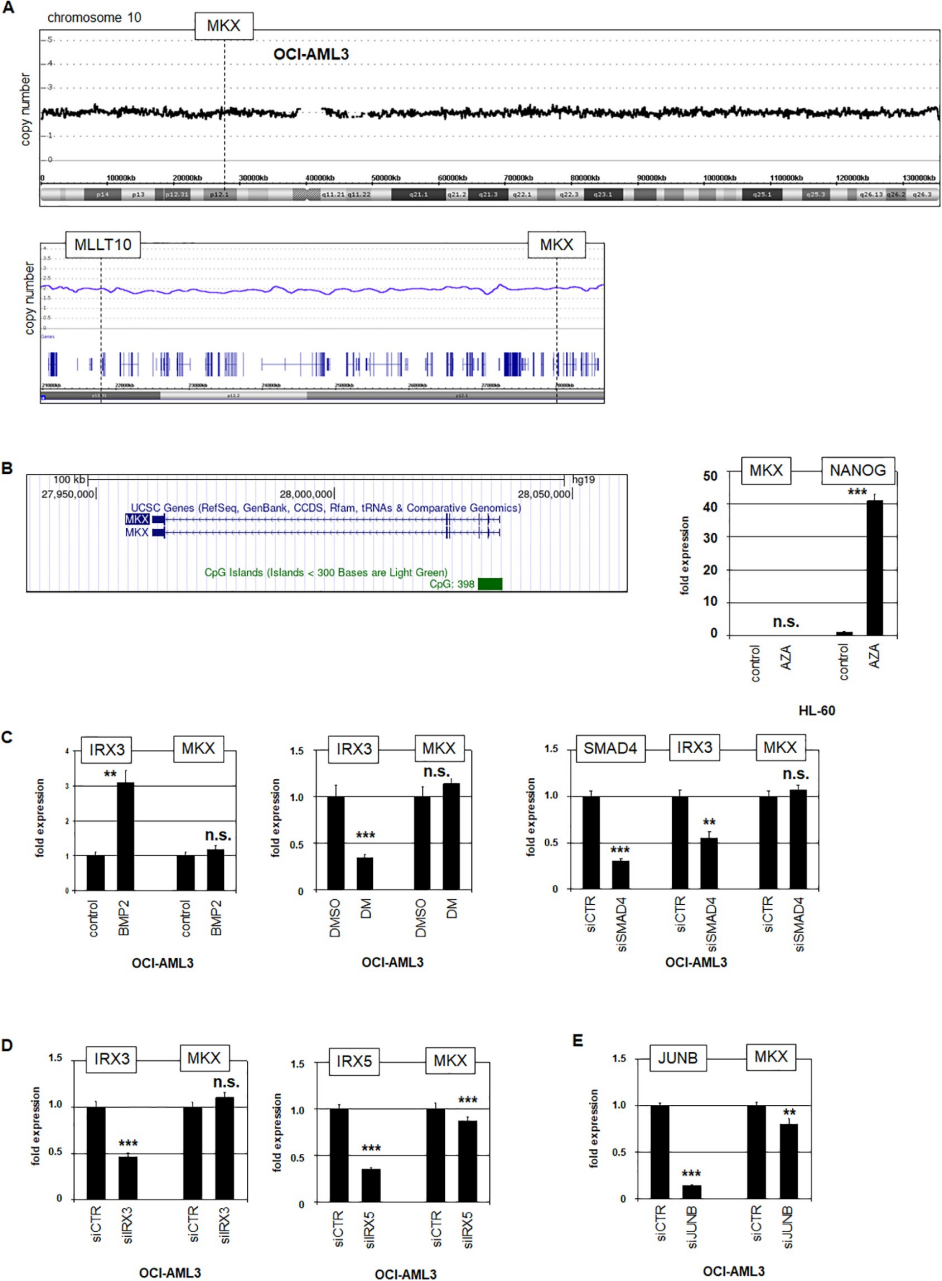
Aberrant regulation of *MKX* in OCI-AML3. (A) Genomic copy number analysis of OCI-AML3 showing the complete chromosome 10 (above), and the chromosomal region 10p12.1-p12.3 highlighting *MLLT10* and *MKX* (below). (B) Data obtained from the UCSC genome browser indicating a CpG-island at *MKX* (left). RQ-PCR analysis of *MKX* and *NANOG* in AML cell line HL-60 treated with DNA-methyltransferase inhibitor AZA (right). The expression level of HL-60 control was set as 1 while the remaining samples are related to that value. (C) RQ-PCR analysis of *IRX3* and *MKX* in OCI-AML3 treated with BMP2 (left) and dorsomorphin (middle). RQ-PCR analysis of *SMAD4*, *IRX3* and *MKX* in OCI-AML3 treated for siRNA-mediated knockdown (right). (D) RQ-PCR analysis of *IRX3* and *MKX* (left), and *IRX5* and *MKX* (right) in OCI-AML3 treated for siRNA-mediated knockdown. (E) RQ-PCR analysis of *JUNB* and *MKX* in OCI-AML3 treated for siRNA-mediated knockdown. The expression level of OCI-AML3 siCTR was set as 1 while the remaining samples are respectively related to that value. Statistical significance was assessed by Student´s T-Test (two-tailed) and the resultant p-values indicated by asterisks (* p<0.05, ** p<0.01, *** p<0.001, n.s. not significant).

Chromosomal rearrangements and/or genomic aberrations frequently underlie gene deregulation in hematopoietic malignancies including AML [39, 40]. However, the reported karyotype from OCI-AML3 discounted conspicuous chromosomal abnormalities at the *MKX* gene located 10p12.1 (www.DSMZ.de). Furthermore, we performed genomic profiling of OCI-AML3, similarly discounting copy number alterations at chromosome 10, including the cryptic del(10)(p12.1-p12.3) which can generate fusion gene *MLLT10*::*MKX*, as described in pediatric BCP-ALL (Fig 2A) [41]. Of note, chromosomal aberration t(10;11)(p12;q23) is a frequent rearrangement in AML which also targets *MLLT10* at position 10p12.3, but generates the fusion gene *KMT2A*::*MLLT10* [42]. Thus, neither chromosomal nor genomic aberrations at position 10p12 were detected in OCI-AML3, such as might contribute to *MKX* activation.

Inspection of the *MKX* locus at the UCSC genome browser (www.genome.cseUCSC.edu) indicated the presence of a CpG island in the promoter region which may underlie its (de)regulated expression (Fig 2B). This hypothesis was supported by recent publications, reporting that DNA methylation at *MKX* and other homeobox genes was detected in solid tumors via a pan-cancer screen, and that OCI-AML3 carries a pediatric AML-associated mutation in *DNMT3A* [43, 44]. However, treatment of AML control cell line HL-60 with DNA methyltransferase inhibitor AZA promoted activation of the accordingly regulated homeobox gene *NANOG* but left *MKX* unchanged (Fig 2B) [45], discounting aberrant DNA demethylation in *MKX* deregulation in AML.

Recently, we reported aberrant expression of TALE-class homeobox genes *IRX3* and *IRX5* in OCI-AML3, showing that elevated BMP2-SMAD4-signalling activates *IRX3* [20]. However, neither treatment with BMP2, inhibition of BMP-signalling by dorsomorphin, nor siRNA-mediated knockdown of *SMAD4* altered the expression of *MKX*, excluding its activation via this pathway in OCI-AML3 (Fig 2C). In contrast, siRNA-mediated knockdown of *IRX5* (but not of *IRX3*) resulted in slightly reduced *MKX* expression levels (Fig 2D). Furthermore, *MKX* expression was also reduced by knockdown of *JUNB* (Fig 2E), which has been shown to play activating roles for *IRX3* and *IRX5* [20]. Together, these findings showed that IRX5 and JUNB support *MKX* activation in OCI-AML3.

**Fig 3:**
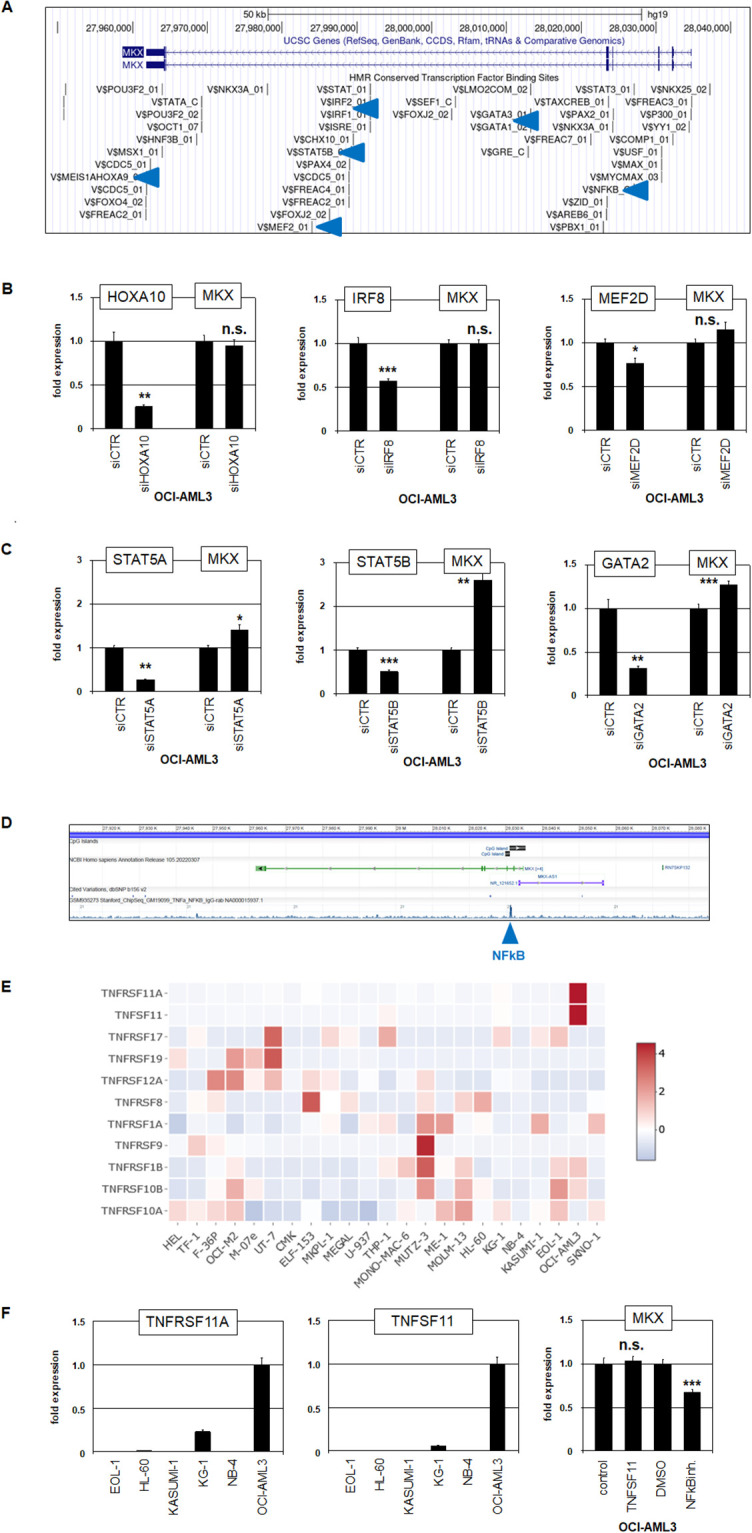
(De)regulation of *MKX* in OCI-AML3. (A) Data obtained from the UCSC genome browser indicating potential TF-binding sites (blue arrow-heads) at the *MKX* locus. (B) RQ-PCR analysis of *HOXA10* and *MKX* (left), *IRF8* and *MKX* (middle), and *MEF2D* and *MKX* in OCI-AML3 treated for siRNA-mediated knockdown. (C) RQ-PCR analysis of *STAT5A* and *MKX* (left), *STAT5B* and *MKX* (middle), and *GATA2* and *MKX* in OCI-AML3 treated for siRNA-mediated knockdown. The expression level of OCI-AML3 siCTR was set as 1 while the remaining samples are respectively related to that value. (D) ChIP-seq data from the ENCODE project showing direct binding of NFkB at the *MKX* locus. (E) Heatmap, based on RNA-seq data, showing gene expression levels from selected NFkB-signalling components, demonstrating elevated activity of *TNFRSF11A* and *TNFSF11* in OCI-AML3. (F) RQ-PCR analysis of *TNFRSF11A* (left) and *TNFSF11* (middle) in selected AML cell lines. RQ-PCR analysis of *MKX* in OCI-AML3 treated with TNFSF11 and NFkB-inhibitor (right). The expression level of OCI-AML3 control was set as 1 while the remaining samples are related to that value. Statistical significance was assessed by Student´s T-Test (two-tailed) and the resultant p-values indicated by asterisks (* p<0.05, ** p<0.01, *** p<0.001, n.s. not significant).

To examine the potential role of other TFs in *MKX* deregulation we screened TF binding sites within its gene sequence using public UCSC genome browser data (www.genome.cseUCSC.edu) as indicated in Fig 3A. We focused on HOXA, IRF, MEF2, STAT5, GATA and NFkB–a panel representing basic regulators of normal and malignant myelopoiesis. We performed siRNA-mediated knockdown of *HOXA10* which, though reportedly regulating *IRX3* and *IRX5* [20], showed no regulatory impact on *MKX* (Fig 3B). *MEF2C* and *MEF2D* regulate tendon and muscle development while *IRF8* and *MEF2D* co-operate oncogenically in AML [46–48]. Furthermore, RNA-seq data from AML cell lines demonstrated highly expressed *IRF8* exclusively in OCI-AML3, indicating aberrant overexpression in this cell line (S4 Fig). However, knockdown of neither *IRF8* nor *MEF2D* altered the expression of *MKX* (Fig 3B), excluding these factors as *MKX* regulators in OCI-AML3. In contrast, siRNA-mediated knockdown of *STAT5A* and *STAT5B* enhanced *MKX* expression (Fig 3C), demonstrating that these factors are transcriptional repressors of *MKX*. Interestingly, RNA-seq data from AML cell lines showed reduced expression levels of both *STAT5A* and *STAT5B* in OCI-AML3 (S4 Fig), thereby supporting *MKX* activity in this cell line. Next, knockdown of myeloid differentiation factor *GATA2* resulted in elevated *MKX* expression, also demonstrating a repressive role (Fig 3C). Similar to *STAT5*, RNA-seq data showed reduced *GATA2* expression in OCI-AML3 (S4 Fig). Finally, we investigated the role of NFkB in MKX regulation. ChIP-seq data from the ENCODE-project demonstrated direct binding of NFkB to the indicated binding site at *MKX* (Fig 3D). RNA-seq data from 23 AML cell lines for selected TNF-signalling components revealed for OCI-AML3 elevated expression of *TNFSF11* and its receptor *TNFRSF11A* which reportedly activate NFkB TFs (Fig 3E) [49]. RQ-PCR analysis confirmed their high expression levels in OCI-AML3 (Fig 3F). While stimulation of OCI-AML3 with additional TNFSF11 spared *MKX* expression in OCI-AML3, treatment with a pharmacological NFkB-inhibitor suppressed *MKX* transcription, showing a significant activating impact of NFkB in *MKX* expression (Fig 3F).

Taken together, we identified IRX5, JUNB and NFkB as activators, and downregulated STAT5A/B and GATA2 as repressors for *MKX* in AML cell line OCI-AML3 which collectively control aberrant expression of this homeobox gene.

### MKX target genes and function in AML

**Fig 4:**
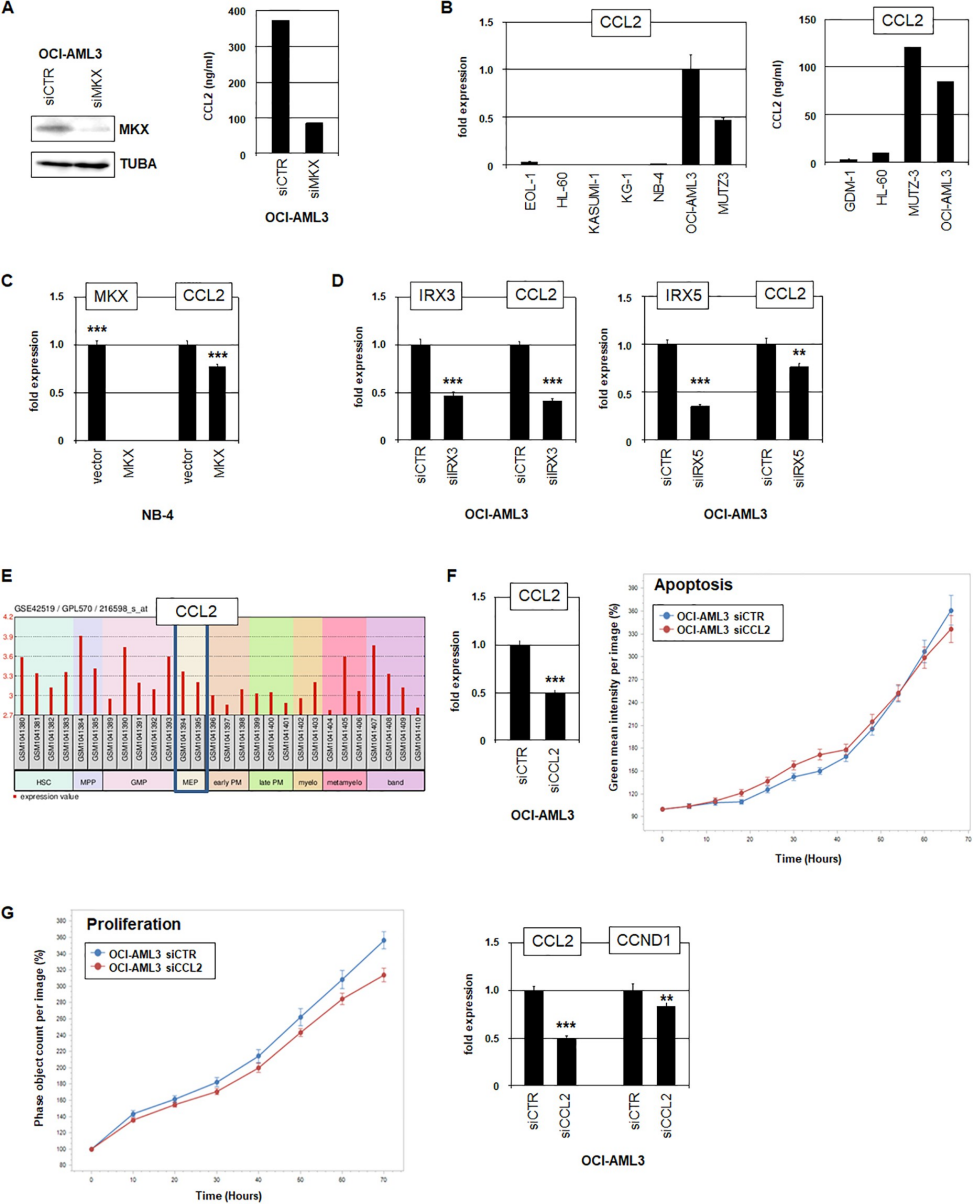
MKX activates CCL2 in OCI-AML3. (A) Western blot analysis of MKX in OCI-AML3 treated for siRNA-mediated knockdown of MKX. TUBA served as loading control (left). ELISA analysis of CCL2 protein in supernatants from OCI-AML3 treated for siRNA-mediated knockdown of *MKX* (right). (B) RQ-PCR analysis of *CCL2* in selected AML cell lines (left). ELISA analysis of CCL2 protein in supernatants from selected AML cell lines (right). (C) RQ-PCR analysis of NB-4 treated for MKX-overexpression resulted in reduced *CCL2* transcript levels. (D) RQ-PCR analysis of *IRX3* and *CCL2* (left), and *IRX5* and *CCL2* (right) in OCI-AML3 treated for siRNA-mediated knockdown. (E) Gene expression profiling data for *CCL2* from hematopoietic stem and developing myeloid cells using public dataset GSE42519. (F) RQ-PCR analysis of *CCL2* in OCI-AML3 treated for siRNA-mediated knockdown of *CCL2* (left). Live-cell imaging analyses of OCI-AML3 treated for siRNA-mediated knockdown of *CCL2*, detecting apoptosis (right) and (G) proliferation (left). The data for the last point of imaging showed no significant difference for apoptosis but for proliferation (p = 0.0059). RQ-PCR analysis of *CCL2* and *CCND1* in OCI-AML3 treated for siRNA-mediated knockdown of *CCL2* (right). The expression levels of NB-4 vector and OCI-AML3 siCTR were set as 1 while the remaining samples are respectively related to those values. Statistical significance was assessed by Student´s T-Test (two-tailed) and the resultant p-values indicated by asterisks (* p<0.05, ** p<0.01, *** p<0.001, n.s. not significant).

To identify target genes regulated by MKX in AML we subjected OCI-AML3 to siRNA-mediated knockdown of *MKX* and subsequent RNA sequencing analysis. After confirming knockdown at transcript and protein levels (Fig 4A), the most significantly expressed genes differing between knockdowns and controls were listed, showing 78 activated and 28 genes repressed by MKX (S1 Table). For more detailed analysis we selected activated *CCL2* (also known as *MCP-1*) which encodes a ligand that binds and activates its cognate receptor CCR2, plays multifaceted roles in myeloid immune cells, and acts as an oncogene in some cancer types [50, 51]. Knockdown of *MKX* in OCI-AML3 was confirmed by Western blot analysis, and the concomitant reduction of CCL2 protein in the supernatant by ELISA analysis (Fig 4A). RQ-PCR analysis showed enhanced *CCL2* expression levels in AML cell lines OCI-AML3 and MUTZ-3, and corresponding expression was detected at the protein level by ELISA (Fig 4B). Thus, *CCL2* is activated by MKX in OCI-AML3 and, accordingly, strongly expressed. However, forced expression of MKX in MKX-negative AML cell line NB-4 reduced *CCL2* transcription (Fig 4C), indicating the absence of additional factors implicated in the regulation of *CCL2* or cooperation with MKX.

Analysis of the *CCL2* promoter region revealed three binding sites for MKX (at -1418 bp, -663 bp, -510 bp) indicating direct regulation (S5 Fig). This region also contained a binding site for IRX factors (at -574 bp) which slightly differ from that for MKX, suggesting transcriptional regulation of *CCL2* by IRX3 and/or IRX5 as well (S5 Fig). Consistently, siRNA-mediated knockdown of *IRX3* and *IRX5* resulted in reduced *CCL2* expression, confirming stimulation by both factors (Fig 4D). Thus, *CCL2* is activated by aberrantly expressed TALE homeodomain TFs MKX, IRX3 and IRX5 in AML cell line OCI-AML3. Of note, *CCL2* is not significantly expressed during early myelopoiesis, indicating stage-independent effects when ectopically activated (Fig 4E). Functional analysis of *CCL2* was performed in OCI-AML3 via its knockdown which was confirmed by RQ-PCR (Fig 4F). Live-cell imaging analysis of the treated cells discounted any role in apoptosis (Fig 4F) but rather that of driving proliferation (Fig 4G). Consistently, siRNA-mediated knockdown of *CCL2* resulted in downregulation of *CCND1*, demonstrating that CCL2 activates this proliferation driver in OCI-AML3 (Fig 4G).

**Fig 5:**
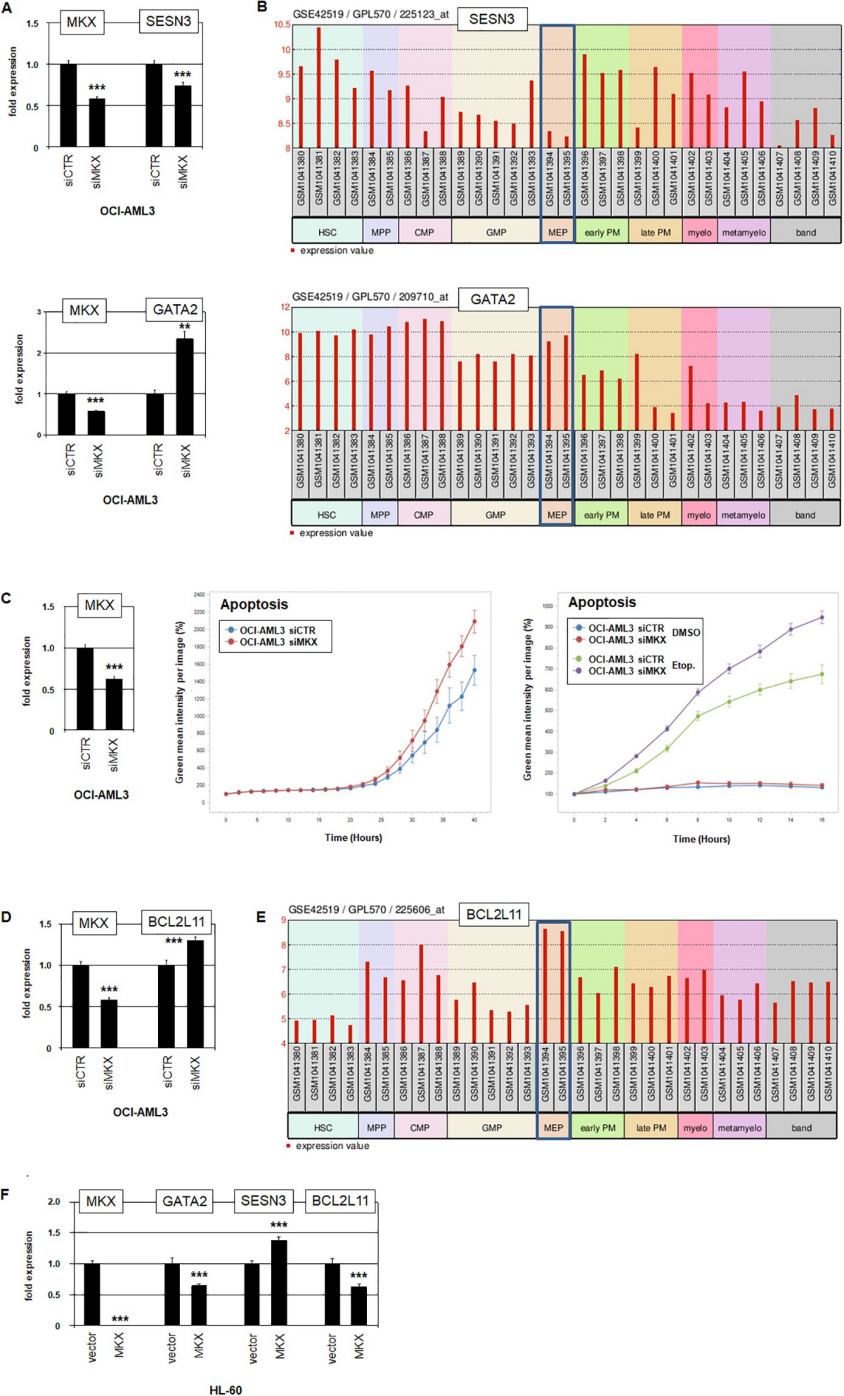
MKX impacts apoptosis and differentiation. (A) RQ-PCR analysis of *MKX* and *SESN3* (above) and of *MKX* and *GATA2* (below) in OCI-AML3 treated for siRNA-mediated knockdown of *MKX*. (B) Gene expression profiling data for *SESN3* (above) and *GATA2* (below) from hematopoietic stem and developing myeloid cells using public dataset GSE42519. (C) RQ-PCR analysis of MKX in OCI-AML3 treated for siRNA-mediated knockdown of *MKX* (left). Analysis of apoptosis by live-cell imaging of OCI-AML3 treated for siRNA-mediated knockdown of *MKX* (middle, p = 0.0052) and additionally with etoposide (right, p = 0.0148). The calculated p-values refer to the last point of imaging. (D) RQ-PCR analysis of *MKX* and *BCL2L11* in OCI-AML3 treated for siRNA-mediated knockdown of *MKX*. (E) Gene expression profiling data for *BCL2L11* from hematopoietic stem and developing myeloid cells using public dataset GSE42519. (F) RQ-PCR analysis of HL-60 treated for MKX-overexpression resulted in reduced transcript levels of GATA2 and BCL2L11, and elevated SESN3 levels. The expression levels of OCI-AML3 siCTR and HL-60 vector were set as 1 while the remaining samples are respectively related to those values. Statistical significance was assessed by Student´s T-Test (two-tailed) and the resultant p-values indicated by asterisks (* p<0.05, ** p<0.01, *** p<0.001, n.s. not significant).

Additional MKX target genes identified by our knockdown/RNA-sequencing approach revealed activated *SESN3*, and repressed *GATA2* (S1 Table). *SESN3* (*SESTRIN 3*) encodes a stress-regulator which reduces intracellular reactive oxygen species (ROS), and *GATA2* a myelopoietic TF [52, 53]. Regulation of these genes by MKX was confirmed by RQ-PCR analysis after siRNA-mediated knockdown (Fig 5A). In MEPs, expression profiling data from developmental stages of myelopoiesis demonstrated reduced levels of *SESN3*, and elevated levels of *GATA2* (Fig 5B). Therefore, deregulation of these genes at that stage may disturb survival and differentiation processes during early myelopoiesis.

Identification of MKX-deregulated survival factor SESN3 prompted analysis of the impact of MKX on apoptosis via knockdown and live-cell imaging. Consistent with our other findings, the data revealed a prominent role for MKX in cell survival (Fig 5C). Moreover, simultaneous treatment with apoptosis-inductor etoposide enhanced the observed *MKX* knockdown effect (Fig 5C). *BCL2L11* encodes a fundamental activator of apoptosis, acts in the context of etoposide, and is regulatory connected with SESN3 [52, 54]. Accordingly, knockdown of *MKX* in OCI-AML3 resulted in *BCL2L11* activation (Fig 5D), indicating that combined activation of *SESN3* and suppression of *BCL2L11* is responsible for the positive role of MKX in cell survival. Moreover, analysis of the promoter region of *BCL2L11* revealed MKX binding sites at -1424 bp and -1291 bp (S5 Fig), suggesting direct transcriptional regulation by MKX. Interestingly, *BCL2L11* is elevated during myelopoiesis at the MEP stage and may, therefore, physiologically predispose these progenitors to apoptosis (Fig 5E). Finally, forced expression of *MKX* in AML cell line HL-60 suppressed *GATA2* and *BCL2L11*, and activated *SESN3* (Fig 5F), confirming the regulatory impact observed in OCI-AML3.

**Fig 6:**
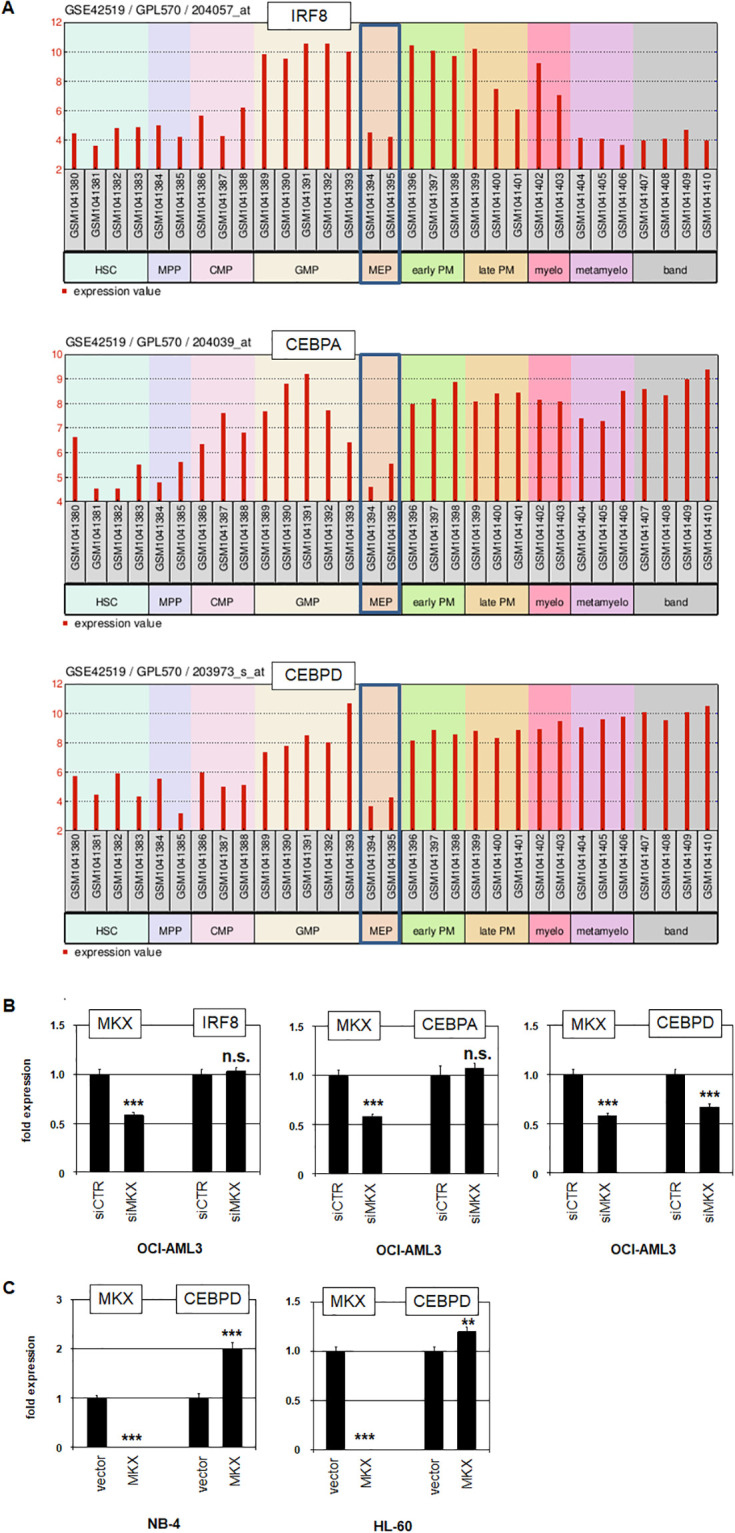
MKX activates *CEBPD*. (A) Gene expression profiling data for *IRF8* (above), *CEBPA* (middle), and *CEBPD* (below) from hematopoietic stem and developing myeloid cells using public dataset GSE42519. (B) RQ-PCR analysis of *MKX* and *IRF8* (left), *MKX* and *CEBPA* (middle), and *MKX* and *CEBPD* (right) in OCI-AML3 treated for siRNA-mediated knockdown of *MKX*. (C) RQ-PCR analysis of NB-4 (left) and HL-60 (right) treated for MKX-overexpression resulted in elevated *CEBPD* transcript levels. The expression levels of OCI-AML3 siCTR, NB-4 vector and HL-60 vector were set as 1 while the remaining samples are respectively related to those values. Statistical significance was assessed by Student´s T-Test (two-tailed) and the resultant p-values indicated by asterisks (* p<0.05, ** p<0.01, *** p<0.001, n.s. not significant).

Next, we analyzed expression profiling data from MM patients comparing *MKX*-high with *MKX*-low samples using public data set GSE19554 and the associated online tool GEOR, which identifies 250 most significantly differing genes. This exercise revealed downregulated *GATA2* and *SESN3* in *MKX*-high MM patients (S6 Fig), showing target genes similar to those identified in AML, although inhibition of *SESN3* contrasts with its activation by *MKX* in OCI-AML3. Furthermore, we found downregulated *CEBPA*, *CEBPD* and *IRF8* in *MKX*-high MM patients (S6 Fig). These three genes are physiologically downregulated in MEPs (Fig 6A), indicating a specific role in myeloid differentiation at that developmental stage. RQ-PCR analysis of these genes in OCI-AML3 after *MKX* knockdown showed that MKX activated *CEBPD* while *IRF8* and *CEBPA* were not regulated by this factor (Fig 6B). Forced expression of *MKX* in NB-4 and HL-60 resulted in elevated *CEBPD* (Fig 6C), confirming MKX-mediated activation of *CEBPD*. Thus, analysis of *MKX* in MM identified *CEBPD* as an additional aberrantly activated target gene in AML.

Taken together, we identified *BCL2L11*, *CCL2*, *CEBPD*, *GATA2* and *SESN3* as MKX target genes in AML cell line OCI-AML3. These genes impact apoptosis, proliferation and differentiation, thus demonstrating several oncogenic functions for deregulated *MKX* in AML. Except *CCL2*, these MKX target genes show conspicuous activity in myelopoiesis at the *IRX1*-positive MEP stage, highlighting this developmental progenitor as a candidate cell of origin in AML cases aberrantly expressing *IRX1*-related *MKX*.

## Discussion

TALE-class homeobox genes encode developmental TFs, regulating basic processes in cell and tissue differentiation [13]. Deregulation of these genes promotes carcinogenesis including leukemia [18, 19, 22]. Accordingly, *IRX1* is physiologically expressed in MEPs and pro-B-cells, while aberrant expression of *IRX2*, *IRX3* and *IRX5* deregulates differentiation processes in AML and BCP-ALL [20, 21, 23]. Hence, these progenitors represent persuasive candidate cells of origin for malignancies aberrantly expressing IRX-related homeobox genes [22]. Here, we show that *MKX* is absent in hematopoiesis and aberrantly activated in AML and MM, mimicking the picture for *IRX2*, *IRX3* and *IRX5* in AML and BCP-ALL, and staging a functional reenactment of the role played by the closely related homeobox gene *IRX1* in early myeloid and B-cell development. Although not in frame, the reported fusion gene *MLLT10*::*MKX* in pediatric BCP-ALL may indicate an oncogenic role for *MKX* in this type of B-cell malignancy as well [41], even though MM is B-cell-derived and rather originates from GC-B-cells [55], contrasting with pro/pre-B-cell originating BCP-ALL. However, this study focused on AML–the role of *MKX* in MM remains unclear and requires additional investigation.

Aberrant expression of *MKX* has also been described in colorectal cancer, suggesting a wider role in carcinogenesis [56]. Physiologically, *MKX* is active in tendon differentiation, inhibiting muscle development via suppression of the master gene *MYOD* [24, 57–59]. MKX-mediated repression of lineage specific regulators occurs via recruitment of co-repressors including SIN3A/HDAC [60]. In tendon development, BMP2-signalling activates *MKX* expression [61], which, however, had no effect in OCI-AML3 cells. In contrast, TNFa-signalling regulates *MKX* expression in tendon as well [62], supporting our discovery of an activating role for TNFa-signalling factor NFkB. OCI-AML3 expresses high levels of *TNFSF11* and *TNFRSF11A* which may control NFkB activation in this cell line.

**Fig 7:**
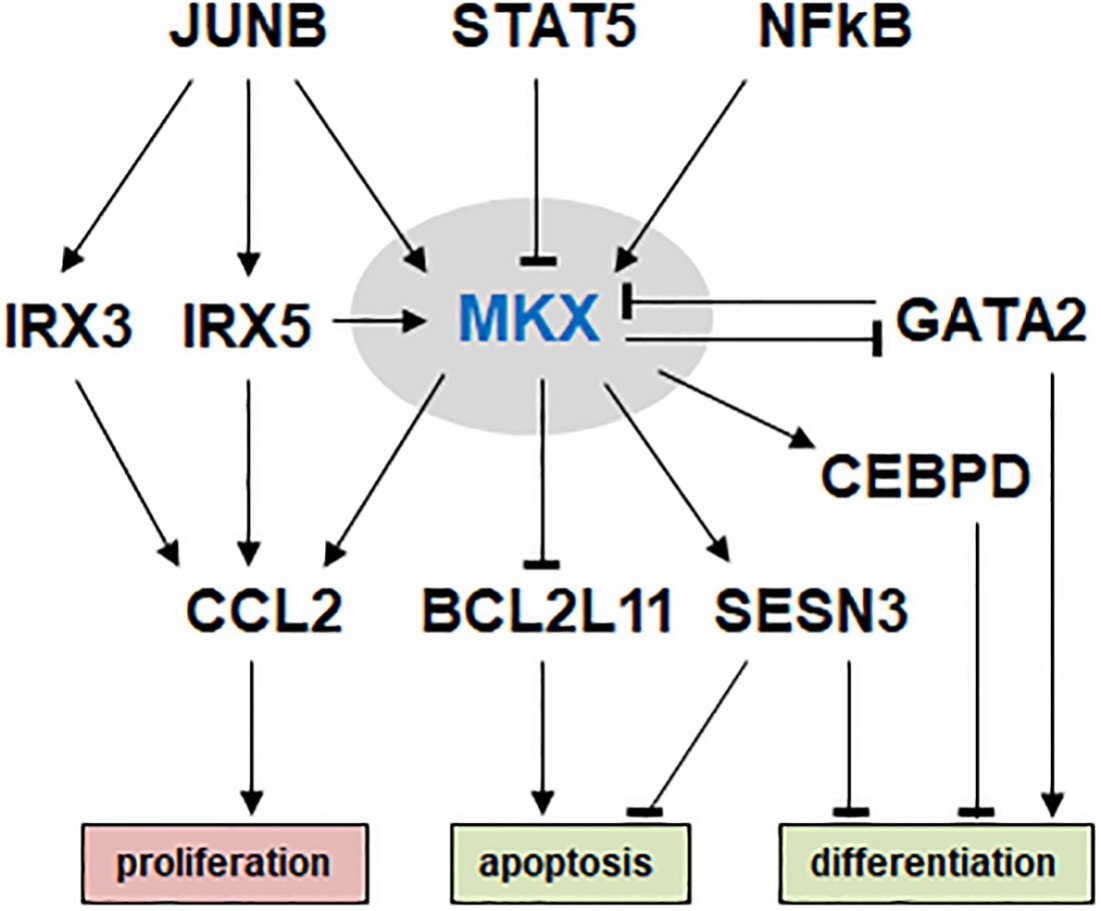
Aberrant gene regulatory network of *MKX* in AML. This diagram summarizes the results of this study. *MKX* is located in the center, regulated by JUNB, STAT5, NFkB, IRX5 and GATA2, and regulates the genes *GATA2*, *CEBPD*, *SESN3*, *BCL11B* and *CCL2*. These deregulated target genes are involved in proliferation, apoptosis and differentiation.

In addition to NFkB, in AML cell line OCI-AML3 we revealed that *MKX* is activated by IRX5 and JUNB, but inhibited by GATA2 and STAT5 (Fig 7). *IRX5* and *JUNB* have been recently described to play an oncogenic role in AML and OCI-AML3 [20]. Our data presented here indicate aberrant downregulation of *GATA2* in OCI-AML3 which in turn supports MKX expression. *GATA2* encodes a basic developmental TF, driving myeloid differentiation. Accordingly, *GATA2* deficiency causes several abnormalities in the myeloid lineage [63]. In contrast, STAT5 inhibits myelopoiesis by repression of *CEBPA* [64]. Consequently, OCI-AML3 expresses elevated *CEBPA* (S4 Fig) and *MKX*. However, the mechanism of *STAT5* downregulation in OCI-AML3 remains to be elucidated.

Our downstream analyses revealed that MKX activates *CCL2* in OCI-AML3. However, forced expression of *MKX* in NB-4 mediated downregulation of *CCL2*, indicating that its transcriptional activation requires additional factors which are absent in NB-4. CCL2 is a secreted ligand and plays together with MKX a role in tenogenic differentiation, indicating aberrant reactivation of this regulatory pathway [24, 65]. In carcinogenesis including AML, CCL2 acts oncogenic by organizing the tumor microenvironment [51, 66, 67]. Furthermore, CCL2 enhances proliferation of AML cells via activation of *CCND1* [68], which was also demonstrated in OCI-AML3. Interestingly, the CCL2-CCR2-pathway activates NFkB in dendritic cell maturation, and both NFkB and AP1 in renal cells [69, 70]. Since we showed that *MKX* is activated by NFkB and AP1-factor JUNB, these data may suggest a self-reinforcing cycle of *MKX* and *CCL2* in AML. However, this hypothesis is speculative and requires experimental examination. Of note, a non-coding mutation or SNP in the upstream region of *CCL2* creates a novel binding site for the TFs PREP1 and PBX2, representing two members of the TALE-class of homeodomain TFs [71, 72]. This SNP is associated with increased *CCL2* expression, and particular inflammatory and liver diseases [73, 74]. Thus, pathological overexpression of *CCL2* is mediated by certain TALE homeodomain TFs albeit via unrelated mechanisms: (i) a specific sequence alteration enabling binding of constitutive PREP1 and PBX2, or (ii) ectopically expressed MKX using a pre-existing binding site.

Furthermore, we showed that MKX activated *SESN3* and inhibited *BCL2L11* in AML cells. Both target genes are part of the FOXO3-network, regulating ROS-mediated apoptosis as described in neuronal cells [52], explaining our observed survival-effect for MKX. Moreover, *BCL2L11* is activated by etoposide treatment, thus driving apoptosis [75]. Both, *BCL2L11* and *SESN3* show contrasting expression levels in MEPs, increasing the potential of apoptosis-induction at this developmental stage. Therefore, aberrant *MKX* activity may curb this seeming apoptotic predisposition, enhancing survival of the tumor cells.

In this study we revealed several genes showing conspicuous activity during myelopoiesis in MEPs. In addition to the reported homeobox gene *IRX1*, *BCL2L11* and *GATA2* are upregulated while *SESN3* and *CEBPD* are downregulated at this developmental stage. In contrast, MKX inhibits *BCL2L11* and *GATA2*, and activates *SESN3* and *CEBPD*. Furthermore, we showed that GATA2 inhibits expression of *MKX*, demonstrating that these factors are mutual repressors. This type of regulatory connection stabilizes the activity of one partner, namely *MKX* in OCI-AML3. On the other hand, repression of *GATA2* may have fundamental consequences for myeloid differentiation as reported for the *GATA2* deficiency syndrome [63]. Finally, a recent study by Sato and colleagues detected *IRX1* mutations in Down syndrome associated myeloid leukemia, and showed that *IRX1* boosts megakaryocyte/erythroid differentiation, highlighting its role in early myelopoiesis [76]. Together, these findings may indicate that aberrantly expressed *MKX* operates oncogenically in MEPs which may, therefore, represent the cell of origin of *MKX*-positive AML tumors.

In conclusion, we identified ectopic expression of TALE-class homeobox gene *MKX* in AML and MM patients and cell lines (Fig 7). *MKX* is closely related to *IRX1* which is physiologically expressed in early myeloid and B-cell development. Our data support the idea that *MKX* as well as *IRX2*, *IRX3* and *IRX5* act oncogenically at least in part by interfering the developmental activity of *IRX1* in both hematopoietic lineages. However, the role of deregulated *MKX* in the mature B-cell malignancy MM remains unclear and deserves further investigation. Nevertheless, our study throws light on normal and abnormal hematopoiesis and pinpoints novel potential markers and/or therapeutic targets for respectively subtyping and treatment of AML and MM patient subsets.

## Supporting information

S1 Fig Public gene expression data for MKX and IRX1. (A) Gene expression profiling data for *IRX1* (above) and *MKX* (below) from hematopoietic stem and developing myeloid cells using public dataset GSE42519. Expression level 3 is indicated by an arrowhead, demonstrating absent expression of MKX in these cells. (B) Gene expression profiling data for *IRX1* and *MKX* from selected hematopoietic cell types using public dataset GSE19599. The cell types MEP and pro-B-cells are indicated by black and blue boxes, respectively. (C) Expression levels of *MKX* in normal human tissue types (above) and mature hematopoietic cell types (below) using RNA-seq data obtained from the Human Protein Atlas. Selected tissues with high and low *MKX* expression are indicated with red and green arrowheads, respectively.
(TIF)

S2 Fig *MKX* expression in AML and MM patients. Gene expression profiling data for *MKX* and *IRX1* in AML patients (above) and for *MKX* in MM patients (below) using public datasets.
(TIF)

S3 Fig MKX expression in AML cell lines. Gene expression profiling dataset GSE59808 contains 32 AML cell lines and shows significant MKX activity just in OCI-AML3.
(TIF)

S4 Fig Gene expression levels in selected AML cell lines. RNA-seq data for selected genes were used to indicate their expression levels in cell lines via DSMZCellDive. OCI-AML3 is indicated by a blue arrowhead.
(TIF)

S5 Fig Potential binding sites for MKX and IRX factors. Potential binding sites for MKX (red) and IRX factors (blue) are indicated in the promoter regions of *CCL2* (above), *BCL2L11* (middle), and *GATA2* (below). The sequences of the first exons are highlighted in bold.
(TIF)

S6 Fig Comparative gene expression profiling analysis of MM patient data. Public gene expression profiling dataset GSE19554 covers MM patients. We used the online tool GEO2R to compare MM patients expressing high *MKX* levels with patients expressing low *MKX* levels. Selected genes showing statistically significant differences are indicated: *MKX*, *GATA2*, *SESN3*, *CEBPA*, *CEBPD* and *IRF8*.
(TIF)

S1 Table RNA-seq data from OCI-AML3 treated for siRNA-mediated knockdown of MKX. The table shows the combined data obtained from three biological replicates, indicating the log2-fold change and the statistical significance. Highlighted are genes selected for detailed analysis in this study, including *MKX*, *CCL2*, *SESN3*, and *GATA2*.
(PDF)
