# Supplementary figures and images for: IRX-related homeobox gene MKX is a novel oncogene in acute myeloid leukemia
Source: PLoS One. 2024 Dec 17;19(12):e0315196. doi: 10.1371/journal.pone.0315196 (PMC11651569; doi:10.1371/journal.pone.0315196)

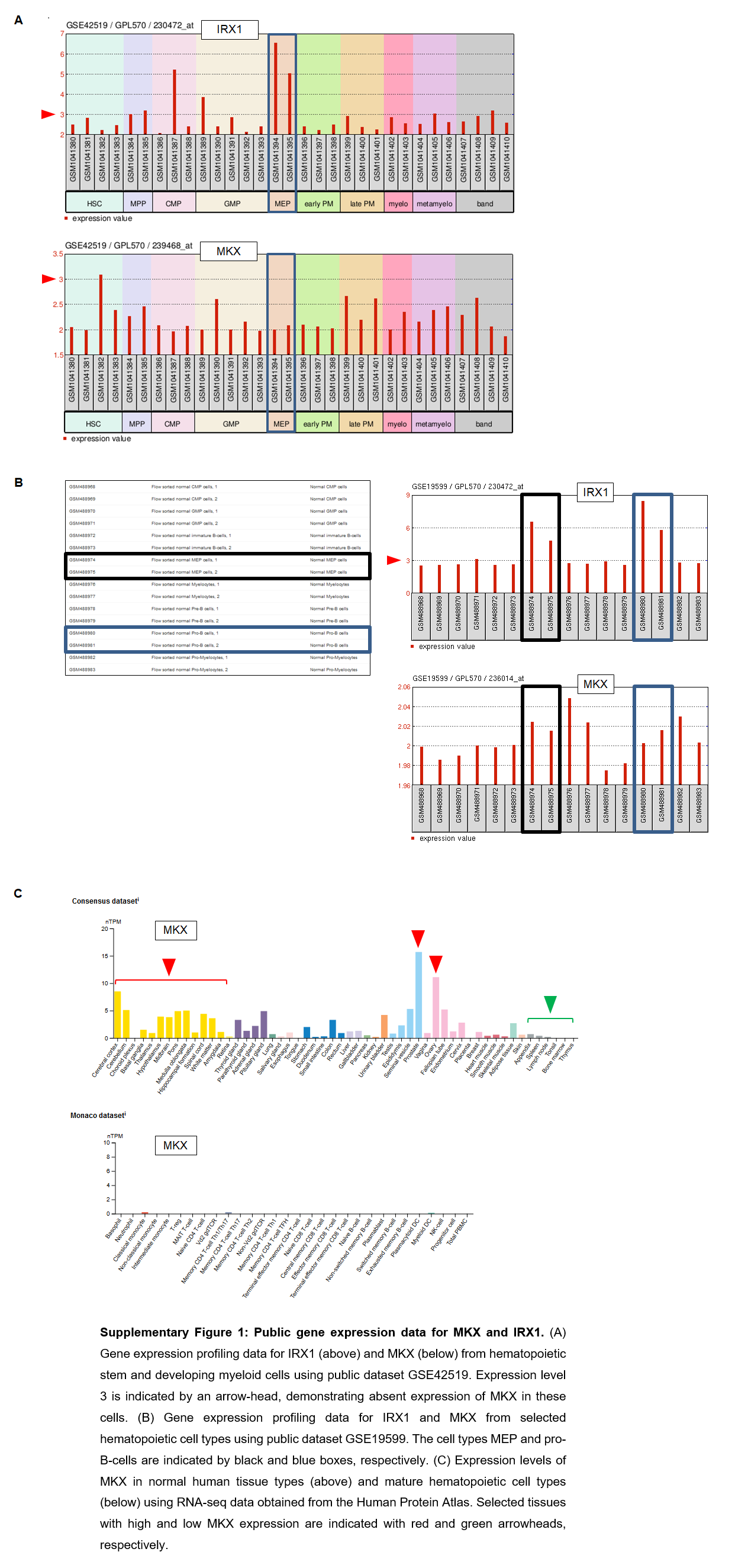

Supplement: S1 Fig — (A) Gene expression profiling data for IRX1 (above) and MKX (below) from hematopoietic stem and developing myeloid cells using public dataset GSE42519. Expression level 3 is indicated by an arrowhead, demonstrating absent expression of MKX in these cells. (B) Gene expression profiling data for IRX1 and MKX from selected hematopoietic cell types using public dataset GSE19599. The cell types MEP and pro-B-cells are indicated by black and blue boxes, respectively. (C) Expression levels of MKX in normal human tissue types (above) and mature hematopoietic cell types (below) using RNA-seq data obtained from the Human Protein Atlas. Selected tissues with high and low MKX expression are indicated with red and green arrowheads, respectively. (TIF) [file pone.0315196.s001.tif]

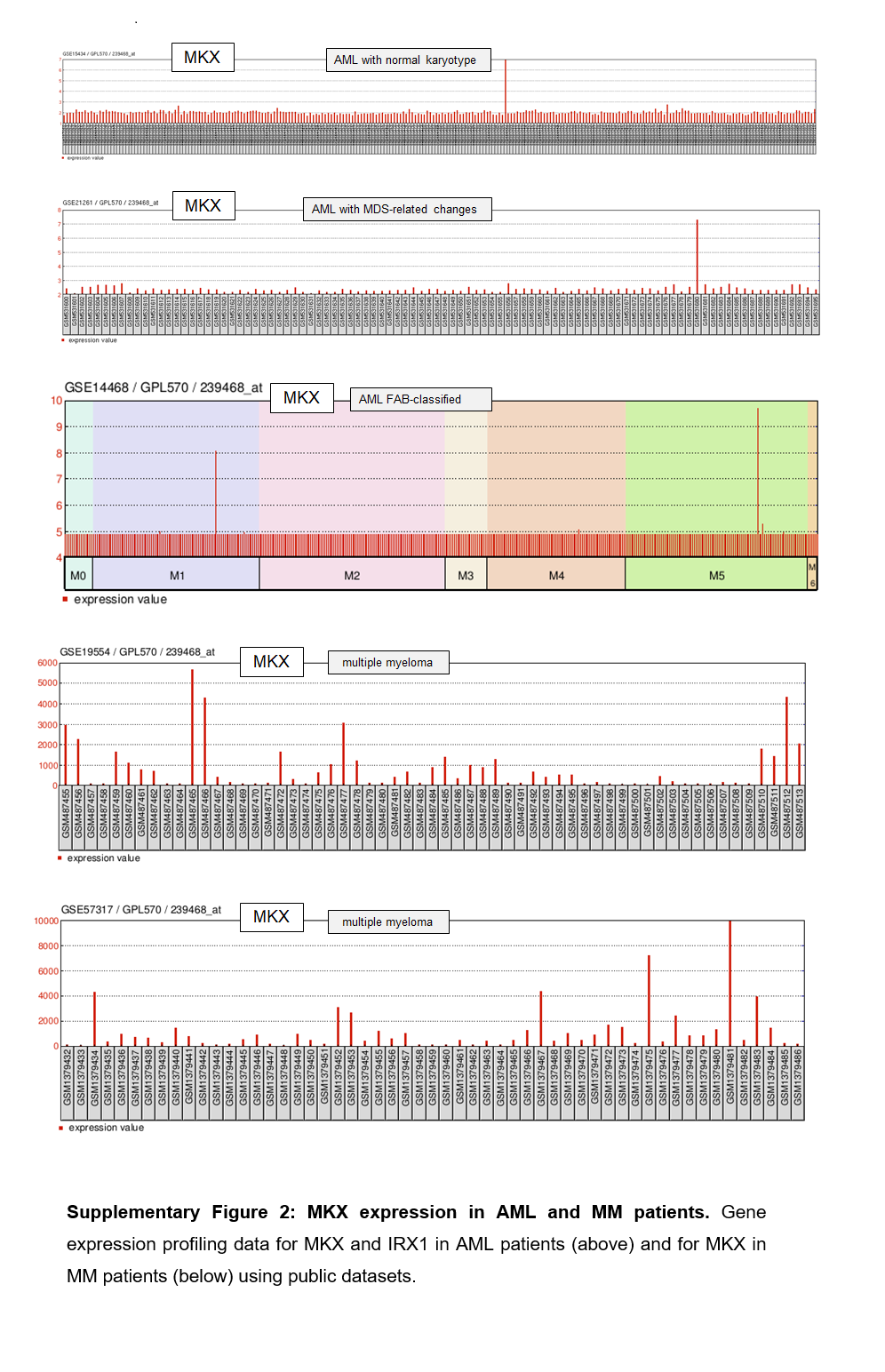

Supplement: S2 Fig — Gene expression profiling data for MKX and IRX1 in AML patients (above) and for MKX in MM patients (below) using public datasets. (TIF) [file pone.0315196.s002.tif]

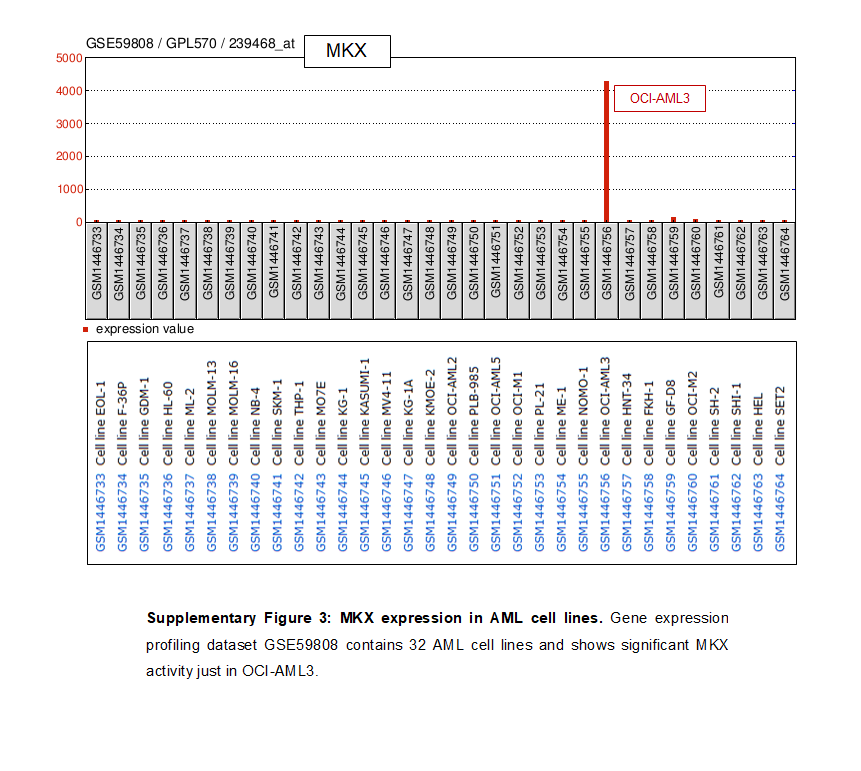

Supplement: S3 Fig — Gene expression profiling dataset GSE59808 contains 32 AML cell lines and shows significant MKX activity just in OCI-AML3. (TIF) [file pone.0315196.s003.tif]

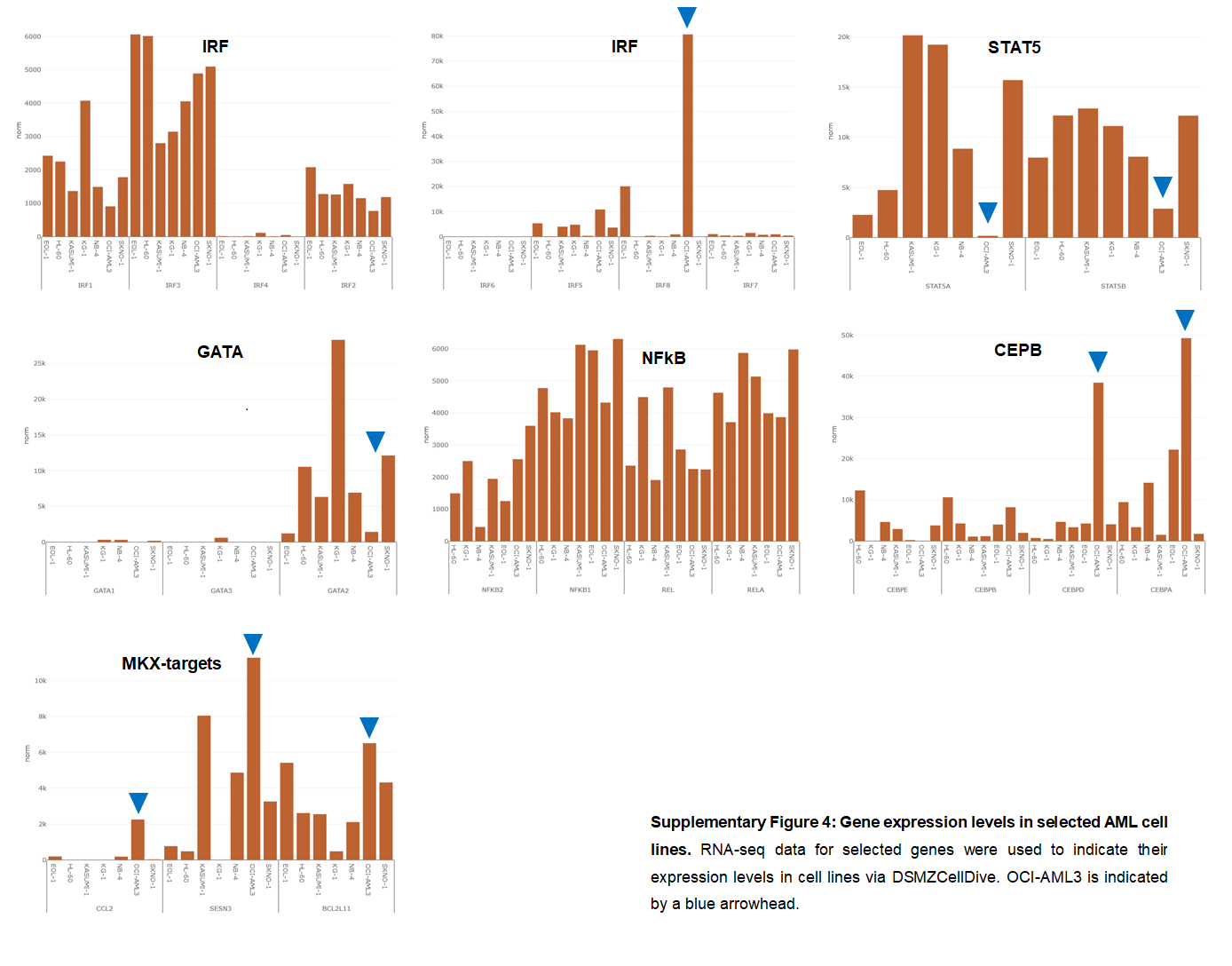

Supplement: S4 Fig — RNA-seq data for selected genes were used to indicate their expression levels in cell lines via DSMZCellDive. OCI-AML3 is indicated by a blue arrowhead. (TIF) [file pone.0315196.s004.tif]

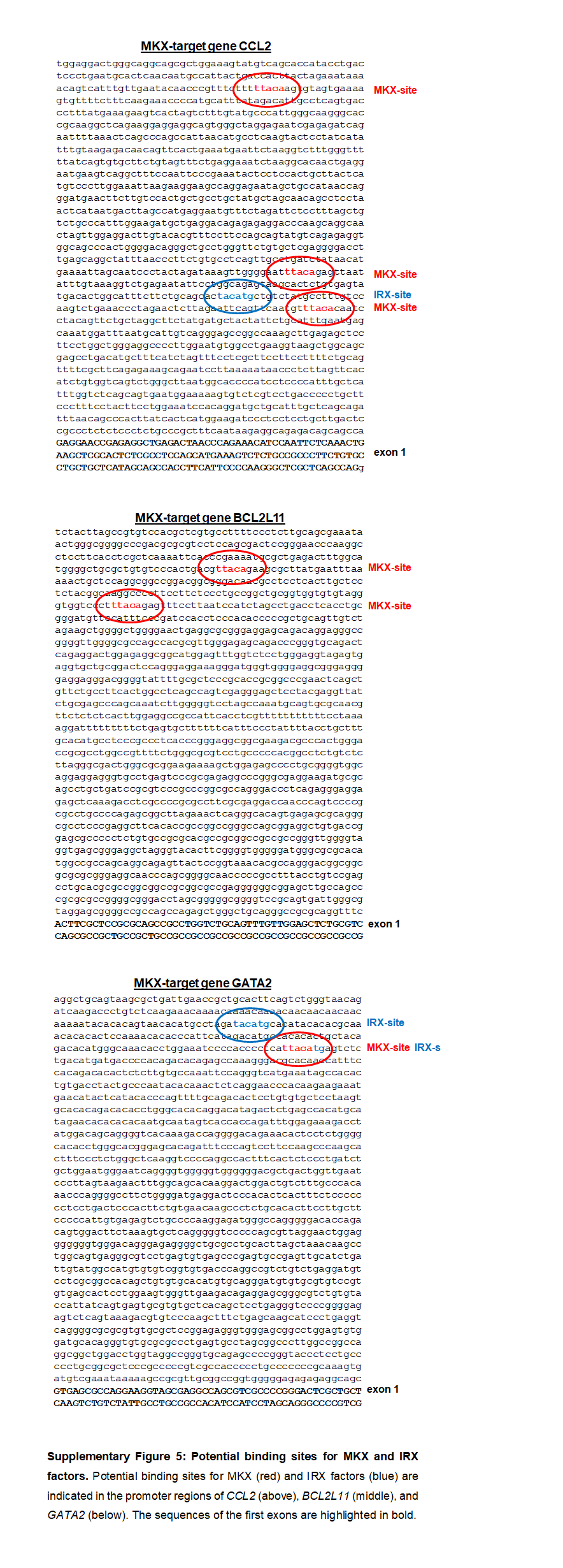

Supplement: S5 Fig — Potential binding sites for MKX (red) and IRX factors (blue) are indicated in the promoter regions of CCL2 (above), BCL2L11 (middle), and GATA2 (below). The sequences of the first exons are highlighted in bold. (TIF) [file pone.0315196.s005.tif]

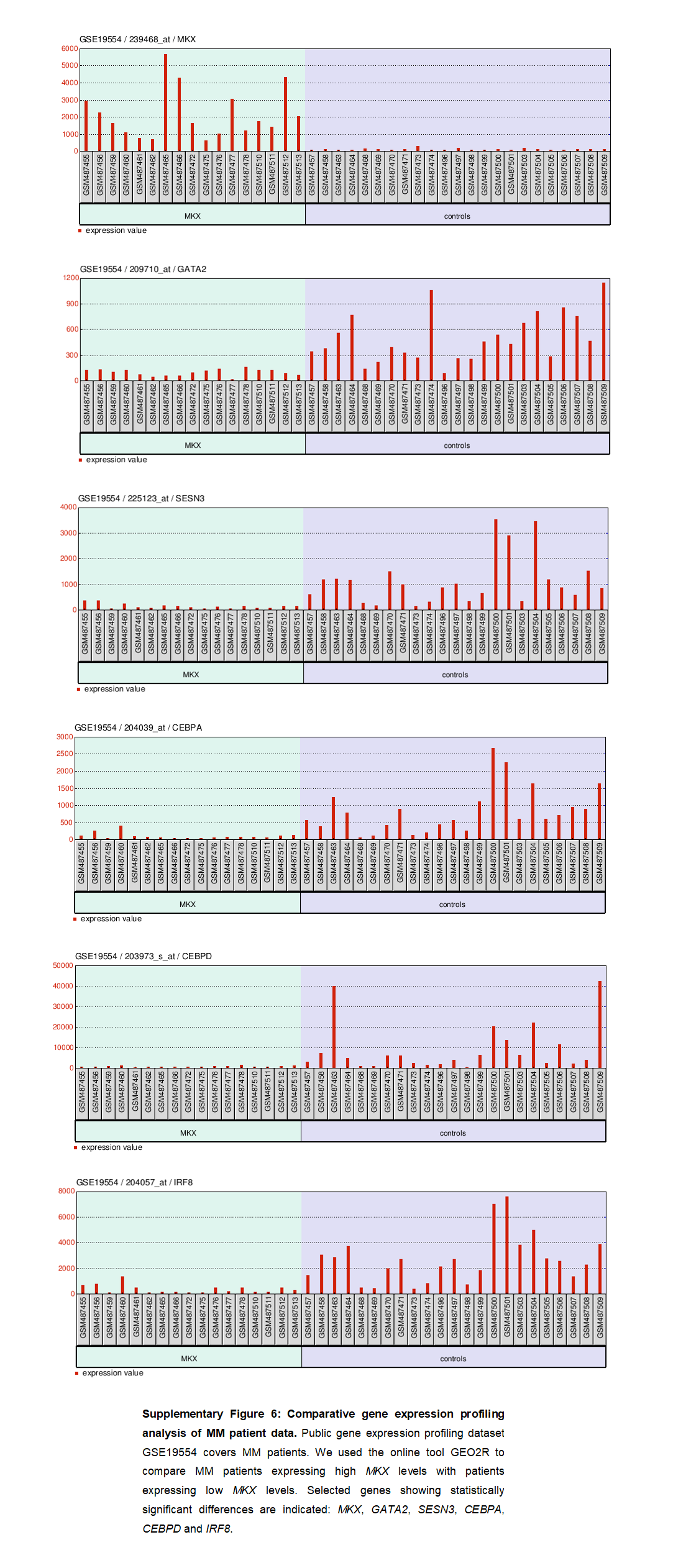

Supplement: S6 Fig — Public gene expression profiling dataset GSE19554 covers MM patients. We used the online tool GEO2R to compare MM patients expressing high MKX levels with patients expressing low MKX levels. Selected genes showing statistically significant differences are indicated: MKX, GATA2, SESN3, CEBPA, CEBPD and IRF8. (TIF) [file pone.0315196.s006.tif]
