# Supplementary material for: IRX-related homeobox gene MKX is a novel oncogene in acute myeloid leukemia
Source: PLoS One. 2024 Dec 17;19(12):e0315196. doi: 10.1371/journal.pone.0315196 (PMC11651569; doi:10.1371/journal.pone.0315196)
Supplement: S1 Table — The table shows the combined data obtained from three biological replicates, indicating the log2-fold change and the statistical significance. Highlighted are genes selected for detailed analysis in this study, including MKX, CCL2, SESN3, and GATA2. (PDF) [file pone.0315196.s007.pdf]

Supplementary Table 1		
**gene**	**log2 fold change**	**padj**
AC008758.1	-8,35	3,67E-07
AC008581.2	-8,23	2,66E-02
EPGN	-4,64	9,26E-02
TNFRSF9	-3,34	6,98E-02
TSHZ2	-3,09	9,91E-02
HP	-1,74	9,83E-02
RGMA	-1,47	6,01E-02
CENPS-CORT	-1,34	6,01E-02
RAB11FIP4	-1,32	2,28E-03
DPYS	-1,30	1,78E-02
NPHP3-ACAD11	-1,28	4,48E-02
**MKX**	-0,96	1,98E-116
LHX1	-0,96	1,28E-02
ZNF235	-0,90	3,48E-02
NAV1	-0,86	2,46E-04
C3AR1	-0,76	5,18E-05
ITGB7	-0,68	7,32E-07
LRRK2	-0,67	9,53E-02
LRRC4	-0,57	1,80E-03
**CCL2**	-0,51	3,67E-07
KCNQ5	-0,51	1,45E-02
ITPR2	-0,51	2,86E-09
DGKH	-0,49	5,86E-02
OLFML2B	-0,48	1,63E-06
PGM2L1	-0,48	2,84E-02
AC006538.2	-0,48	9,91E-02
NBPF20	-0,46	6,01E-02
DGKE	-0,46	8,83E-02
MFSD8	-0,42	4,00E-02
ELMO1	-0,41	9,08E-03
UTRN	-0,40	8,54E-04
SYNC	-0,40	7,43E-02
PPBP	-0,40	5,86E-02
MINDY3	-0,37	2,33E-02
ABCA1	-0,37	9,91E-02
LILRB2	-0,35	5,85E-02
**SESN3**	-0,35	4,07E-08
SGK3	-0,34	6,38E-03
DDHD2	-0,34	4,33E-02
SMN2	-0,33	1,33E-04
BRCA2	-0,33	3,11E-02
KSR1	-0,33	6,15E-02
TLNRD1	-0,33	2,36E-02
FAM172A	-0,32	6,98E-02
WDR75	-0,32	2,56E-02
DAB2	-0,32	3,12E-02
VAV3	-0,32	9,26E-02
KPNA3	-0,31	4,11E-06
S100A9	-0,31	1,36E-02
CR1	-0,31	1,37E-03
NPIPB5	-0,31	8,99E-02
BRCA1	-0,30	2,03E-03
ZNF766	-0,30	6,08E-02

HERC1	-0,29	3,58E-02
TRIM59	-0,29	1,13E-02
DCUN1D4	-0,29	6,01E-02
DOCK7	-0,28	1,78E-02
METTL8	-0,28	3,52E-02
NCF2	-0,28	4,33E-02
SLC1A3	-0,28	2,12E-03
NAIP	-0,28	3,72E-07
MCTP1	-0,28	1,40E-02
ERCC6L2	-0,27	2,02E-02
CREBL2	-0,27	2,82E-02
GPR183	-0,27	8,09E-02
MMP9	-0,27	4,32E-04
ONECUT2	-0,26	8,83E-02
ARHGAP19	-0,26	6,79E-04
TMEM229B	-0,26	5,70E-02
KLF7	-0,26	2,04E-02
SACS	-0,26	9,48E-04
UEVLD	-0,26	3,11E-02
AKAP13	-0,25	4,12E-03
RPS10-NUDT3	-0,25	8,72E-02
HUWE1	-0,25	8,52E-02
CRK	-0,25	2,06E-03
TBK1	-0,25	2,06E-03
CBX5	-0,25	2,82E-03
SLC7A6OS	0,25	4,33E-02
BARX1	0,25	5,50E-02
NUP214	0,25	9,26E-02
BMP2	0,26	1,20E-06
FKBP7	0,27	8,95E-02
SPATA6	0,27	9,91E-02
UBXN6	0,27	4,33E-02
ALOX5AP	0,28	1,72E-04
**GATA2**	0,29	3,19E-02
TIMP3	0,29	2,73E-05
EIF5A2	0,29	5,59E-02
CEP41	0,30	7,46E-03
ADAMTS1	0,32	8,13E-08
FN1	0,33	1,25E-18
DZIP1	0,33	6,07E-02
APOL3	0,34	8,84E-02
ART3	0,35	5,69E-03
ABCA6	0,39	8,11E-02
ATF7IP2	0,39	9,87E-03
PJA1	0,40	1,83E-02
ABHD17C	0,43	6,83E-11
TCEA2	0,43	7,43E-02
PPARA	0,43	1,53E-02
SCHIP1	0,44	8,79E-04
METTL7A	0,49	3,31E-04
PCGF2	0,77	1,53E-02
KBTBD3	1,06	1,78E-02
ME3	1,66	4,33E-02

Supplementary Table 1: RNA-seq data from OCI-AML3 treated for siRNA-mediated knockdown of MKX. The table shows the combined data obtained from three biological replicates, indicating the log2-fold change and the statistical significance. Highlighted are genes selected for detailed analysis in this study, including *MKX*, *CCL2*, *SESN3*, and *GATA2*.
